# Relational and interactional dynamic network data from Czech lower-secondary school students

**DOI:** 10.1016/j.dib.2023.109641

**Published:** 2023-10-04

**Authors:** Tomáš Lintner, Klára Šeďová, Martin Sedláček, Zuzana Šalamounová, Roman Švaříček, Karolína Malíková, Ivo Rozmahel, Jakub Vlček, Barbora Nekardová

**Affiliations:** a Department of Educational Sciences, Masaryk University, Arna Nováka 1/1, 602 00 Brno, Czechia; b Institute SYRI, Žerotínovo nám. 617/9, 601 77 Brno, Czechia; c netanlab, Bratislava, Slovakia; d Institute for Research and Development in Education, Charles University, Myslíkova 208/7, 110 00 Prague, Czechia

**Keywords:** Social network analysis, Dynamic social network analysis, Peer relationships, School networks, Classroom group work, Classroom interaction

## Abstract

This article introduces a network data set on the friendships and group work interactions among a convenience sample of 276 Czech Grade 6 students from twelve classrooms, supported by student-level demographic, literacy, motivation, and classroom communication data. Gathered longitudinally at the beginning and end of the 2021/2022 school year, the data provide a relational insight into the nature and evolution of early adolescents' friendships. Moreover, the data provide a unique relational and temporal insight into the verbal interaction of students during classroom group work. This dataset constitutes a valuable resource for educational researchers interested in studying classroom group work as well as for social network scientists studying dynamic social networks.

Specifications Table

**Table:** 

Subject	Social sciences, Education
Specific subject area	Social network analysis
Type of data	Tables
How the data were acquired	The relational data, denoting friendship ties between the students, were gathered through a sociometric questionnaire. Interactional data from group work was obtained via video recordings, which were subsequently transcribed into text and coded using a coding scheme. Both the sociometric questionnaire and the coding scheme are available in the linked dataset. Similarly, data on whole-classroom discussions were collected through video recordings of the lessons. Individual student attributes were derived from a variety of methods. Gender and socioeconomic status (SES) data were collected using a questionnaire, followed by a coding process based on the International Socio-Economic Index of Occupational Status (ISEI) [1] and a three-class version of the European Socio-economic Classification (ESeC) [2,3]. Literacy data were gathered using a standardized reading literacy test by SCIO company [4], and data on achievement motivation were obtained through a validated questionnaire [5].
Data format	Raw
Description of data collection	We collected data from 276 Grade 6 students in twelve classrooms from six comprehensive lower-secondary schools in the South Moravian Region in the Czech Republic. The classrooms were selected as a convenience sample based on the willingness of school directors and teachers to allow data collection.
Data source location	· Region: South Moravian Region · Country: Czech Republic
Data accessibility	Repository name: Mendeley Data Data identification number: 10.17632/9c3dth6cwp.2 Direct URL to data: https://doi.org/10.17632/9c3dth6cwp.2

## 1 Value of the Data

• The dataset provides a relational insight into the friendships of early adolescents and the development of their friendships across the school year. Additionally, the dataset offers a unique relational and temporal insight into the verbal interaction of students during classroom group work.
• These data therefore allow to study the relationship between the interaction in group work and the friendships of students.
• The dataset may be of use to educational researchers interested in studying classroom group work as well as to social network scientists studying dynamic social networks.
• Specifically, the data we provide can be used to study processes influencing friendships and interaction during classroom group work among elementary school students.
• The complex and multilevel structure of the data make them suited for the use of advanced statistical models.
• Also, it provides a valuable resource for testing the viability of emerging dynamic network models on real-world data.

## 2 Objective

The aim of this dataset is to provide an opportunity to educational researchers and social network scientists to study dynamic relational and interactional data from lower-secondary school students. The dataset further aims to contribute to a good practice in open data and reproducibility of research in the field of social network analysis.

## 3 Data Description

The dataset comprises six separate files. Table 1 shows the characteristics of the files. Three xlsx tables contain data on student attributes, relationship data, and interactional data. Three PDF files contain the original Czech version of the sociometric questionnaire, a translated English version of the sociometric questionnaire, and a codebook used for coding interactions. We now describe the individual parts of the dataset along with providing basic descriptive statistics of the collected data.

**Table 1:** List of files.

file name	file format	file content
student attributes	xlsx	A single Excel sheet containing student-level attributes.
relational data	xlsx	24 Excel sheets containing adjacency matrices of friendship nominations at the start and at the end of the school year.
interactional data	xlsx	62 Excel sheets representing time-stamped data of interaction between the students in group work.
sociometric questionnaire - Czech version	pdf	An original Czech version of the sociometric questionnaire.
sociometric questionnaire - English version	pdf	A translated English version of the sociometric questionnaire.
codebook	pdf	A codebook used for the coding of the interactions in group work.

### 3.1 student attributes.xlsx

student attributes.xlsx file contains a single sheet with student-level variables. The following variables are included: *studentID, classroomID, classroom_type, gender, EseC, HISEI, whole_classroom_talk_start, whole_classroom_talk_end, literacy_start, literacy_end, need_of_success*, and *avoidance_of_failure. studentID* is a unique student identifier consisting of four numbers – the first two numbers make up the *classroomID*, and the last two numbers denote the student's unique number within the classroom (e.g., 0102 denotes that a student is from classroom 01 and the student's unique number within that classroom is 02). *classroom_type* is a categorical variable denoting whether the classroom underwent an intervention aimed at improving students’ classroom talk (“experimental”) or not (“control”). The intervention was performed during the school year and consisted of a research team working with teachers on how to engage students in whole classroom talk more and how to engage them equally. The intervention is described in more detail in “3. Experimental design, materials and methods” section and here [6]. The interactional data were collected only for the experimental classrooms. *gender* is a categorical variable that can take on two values – “girl” and “boy”. *ESeC* is a categorical variable denoting student's family socioeconomic status according to the three-class version of the European Socio-economic Classification [2,3] based on parents’ occupations. “salariat” are large employers, professionals, managers, and supervisory occupations – usually requiring university-level education – “intermediate” encompass small employers, self-employed, higher-grade white collar, and higher-grade blue-collar occupations, “working_class” encompass lower and routine service, sales, clerical, and manual occupations. *HISEI* also denotes student's family socioeconomic status based on parents’ occupations, however, it is an integer in the range of 16 to 90 based on higher of the parents’ International Socio-Economic Index of Occupational Status [1]. *whole_classroom_talk_start* and *whole_classroom_talk_end* denote how much student talked during regular lessons at the start and at the end of the school year measured in seconds per lesson. *literacy_start* and *literacy_end* denote the level of literacy of a student at the start and at the end of the school year measured by a standardized Czech language literacy test by SCIO [4] as a continuous variable from -100 to 100. A student would get -100 points if they answered all items incorrectly, 0 points if they did not answer any item, and 100 if they answered all the items correctly. *need_of_success* and *avoidance_of_failure* denote student's achievement motivation based on two dimensions by [5] measured as an integer in the range of 1 to 5 with higher values indicating higher need of success and higher avoidance of failure. Table 2 shows basic descriptive statistics of the student-level variables.

**Table 2:** Descriptive statistics of student-level variables.

variable	N (%)	M (SD)	missing N (%)
*classroom_type*			0 (0.00%)
experimental	145 (52.54%)		
control	131 (47.46%)		
*gender*			0 (0.00%)
male	142 (51.45%)		
female	134 (48.55%)		
*ESeC*			31 (11.23%)
salariat	109 (39.49%)		
intermediate	80 (28.99%)		
working_class	56 (20.29%)		
*HISEI*		55.67 (16.36)	31 (11.23%)
*whole_classroom_talk_start*		12.39 (15.69)	34 (12.32%)
*whole_classroom_talk_end*		15.99 (17.09)	12 (4.35%)
*literacy_start*		40.60 (23.34)	55 (19.93%)
*literacy_end*		47.26 (23.06)	40 (14.49%)
*need_of_success*		3.17 (0.91)	74 (26.81%)
*avoidance_of_failure*		2.99 (1.16)	73 (26.45%)

### 3.2 relational data.xlsx

*relational data.xlsx* file contains data on student friendships. The file contains 24 sheets, each sheet containing one adjacency matrix denoting directed friendship nominations between the students in the individual classrooms at the start and at the end of the school year. The sheets are named by *classroomID* followed by “start” or “end” suffix denoting whether the matrix represents friendships at the start or at the end of the school year (e.g., 01start containing matrix for classroom 01 at the start of the school year). The first column and row in each sheet contains ordered *studentID* variables representing the individual students. The values in the matrix take on “1”, “0”, or “NA” with “1” representing the existence of the friendship nomination tie from one student to another, “0” representing no friendship nomination tie from one student to another, and “NA” representing missing values. The relational data contain 12,764 ties in total, 1,053 (8.25%) of them are missing.

### 3.3 interactional data.xlsx

*interactional data.xlsx* file contains data on verbal interaction between the students during group work. The file contains 62 sheets, each sheet containing one time-ordered and time-stamped list of verbal interactions between two or more students during one group work session. Each experimental classroom undertook group work sessions two times during the school year. The sheets are named by *classroomID* followed by a letter denoting when the group work session happened with “a” denoting first time and “b” denoting second time group session was performed, and another letter denoting the specific group within the classroom (e.g., 01aa containing data on interaction from the first group session from classroom 01 from group “a”). Each sheet contains four columns named *sender, receiver, increment*, and *time. sender* contains the *studentID* of student making an utterance, *receiver* contains the *studentID* of student being the target of the *sender* student utterance, *increment* denoting a change of the relational event from its past value, and *time* shows when the interaction occurred measured in seconds from the first utterance in the group. In total, the interactional data contain 4721 interactional events – on average, 76.15 events per group work session.

### 3.4 Supporting pdf files

*sociometric questionnaire - Czech version.pdf* file contains the original Czech version of the sociometric questionnaire used for the generation of the adjacency matrices with *sociometric questionnaire - English version.pdf* file containing an English translated version of the questionnaire. *codebook.pdf* file contains a codebook used during the coding process of the interaction during group work.

## 4 Experimental Design, Materials and Methods

The data we provide were collected for a research project called *Collectivity in dialogic learning: an interventional study*. The main aim was to determine whether an intervention led by researchers among comprehensive lower-secondary class teachers could enhance classroom communication and subsequently improve student performance. The intervention consisted of a series of group workshops led by our research team focusing on aspects of dialogic learning and a series of one-to-one meetings between the individual researchers and the individual teachers discussing communication patterns of the individual students. Although the main aim of the project did not constitute any relational data, we made every effort to collect as much relational data as possible believing that student relationships played part in classroom communication, student performance, as well as interaction in group work.

Due to the resource-intensive nature of the project, we worked with a limited sample comprising six experimental and six control classrooms. We selected only comprehensive lower-secondary schools in the South Moravian Region of the Czech Republic to ensure high comparability among the classrooms. As a result, we did not include any selective or special schools. The classrooms were chosen as a convenience sample, based on the willingness of school directors and teachers to allow data collection. Since the project required intense collaboration between the research team and the teachers, we included only those teachers who were open to this level of cooperation. The use of a convenience sample and the narrow geographical focus might limit the generalizability of future analyses. Thus, our data is better suited for studying the microprocesses within classrooms and assessing the suitability of dynamic network models on real-world data, rather than for broader educational settings.

We employed several methods to collect our data – pen and paper questionnaires, computer-based questionnaires, and recorded classroom observations followed by a coding process.

The relational data denoting friendship ties between the students were acquired with a pen-and-paper sociometric questionnaire consisting of a single nomination question [7,8] worded as ‘Write the names of the classmates you are friends with. You can write as many names as you want. The order of the names does not make any difference.’ A trained researcher administered the questionnaire in group settings in the classrooms during school lessons and provided the participants with the necessary assistance. We drew from previous research, which aimed to identify friendship ties between classmates (e.g., [9]), to formulate the nomination question. Additionally, we consulted a linguistic expert when crafting the question in the Czech language.

The interactional data from group works were acquired with video recordings of the group works followed by a transcription into text and a coding process. The full coding scheme used to create the interactional data is available in the linked dataset. We coded only interaction based on on-task communication – that is communication relating to the task the group is working on – it included substantive communication relating to solving the task, but also communication around organizing students’ workload. We did not distinguish between the different types of interaction – e.g., questions, answers, comments – as long as it was an on-task communication.

The individual student attributes were acquired with a combination of different methods. The data on students’ gender and socioeconomic status (SES) were acquired with a computer-based questionnaire with first question asking students to write their gender and two questions worded as “What is the profession of your mother?” and “What is the profession of your father?” followed by a coding process based on International Socio-Economic Index of Occupational Status (ISEI) [1] and a three-class version of the European Socio-economic Classification (ESeC) [2,3] aiming to identify student's socioeconomic status based on their parents’ highest occupational status. To ensure efficient data collection, we used a measure of SES which didn't require parental responses and was quick for students to answer. Consequently, this SES measure might not be the most accurate since it does not explicitly account for parental education and income. Students’ level of literacy was measured with a standardized computer-based reading literacy test by SCIO company [4]. SCIO performs nationwide ability tests in the Czech Republic regularly. Our test contained items from the nationwide testing and was developed in such a way that at both timepoints, the difficulty of the test was the same while none of the test items repeated. Each test contained 26 items covering five areas of literacy – distinguishing between opinions and judgements, distinguishing between subjective and objective statements, identifying manipulative communication in mass-media, using text as a study resource, and forming new text. Our specific tests did not undergo item response theory (IRT) testing; however, our tests were made from the items from the nationwide testing which were formed on IRT, and our specific tests underwent pilot testing. The data on achievement motivation were acquired with a computer-based validated questionnaire, which followed the testing part [5].

Lastly, the data on whole-classroom talk were acquired with video recordings of the lessons. We recorded two consecutive Czech language lessons in each classroom in the beginning and in the end of the 2021/2022 school year, assessed the length of each student's on-task communication in seconds with the help of EduCoM – a specialized mobile application designed for bulk collection of educational communication data in classrooms [10], and averaged the students’ communication across the two consecutive lessons. We collected friendship data along with most individual student-level data with the exception of achievement motivation in two time points – in the beginning and in the end of the school year. We collected data on student achievement motivation in one time point – in the beginning of the school year. The interactional data from group works were collected in the middle of the school year.

## Ethics statements

We obtained oral consent from school principals and teachers and written consent from teachers and parents of participating students. Participants were able to withdraw from research at any time. We also obtained permission for video and audio recordings, which were anonymized prior to analysis. As per the Czech Science Foundation requirements, we did not seek ethical board approval for this project as it was not required.

## CRediT authorship contribution statement

**Tomáš Lintner:** Conceptualization, Methodology, Formal analysis, Investigation, Data curation, Writing – original draft, Visualization. **Klára Šeďová:** Conceptualization, Writing – review & editing, Supervision, Project administration, Funding acquisition. **Martin Sedláček:** Conceptualization, Investigation. **Zuzana Šalamounová:** Conceptualization, Investigation. **Roman Švaříček:** Conceptualization, Investigation. **Karolína Malíková:** Investigation. **Ivo Rozmahel:** . **Jakub Vlček:** Investigation. **Barbora Nekardová:** Validation, Investigation.

## Data Availability

Relational and interactional dynamic network data from Czech lower-secondary school students (Original data) (Mendeley Data).
